# The Value of Ether in Collapse

**Published:** 1883-11

**Authors:** Herbert H. Meyers


### The Value of Ether in Collapse.

I gather from Mr. Caiger's letter in *The Lancet* of August 25th, that the value of ether in collapse is somewhat of a new discovery. I was not aware of the fact when I used it eighteen months ago in a severe case of flooding caused by placenta prævia, the particulars of which I append. I received a message from a fellow practitioner who was ill, asking me to attend a midwifery case for him. On arriving at the house, bag in hand, I found the woman on the bed in such a state that for a moment or two it was doubtful whether she was alive or dead. However, she was alive, and I immediately injected half a drachm of ether. Its effect was magical, so I gave another injection of the same quantity. After this ether and Bonjeau's ergotin were injected together into the buttock, this produced contraction of the uterus, and brought the head down to close the os, where it was maintained in position by abdominal pressure. Shortly afterwards I turned and delivered, and the woman made an excellent and rapid recovery.—*Herbert H. Meyers, M. R. C. S., L. R. C. P., in The Lancet.*

